# Trophoblast Extracellular Vesicles Rewire Immunity: A Symptom‐Relief Avenue for Recessive Dystrophic Epidermolysis Bullosa

**DOI:** 10.1002/jev2.70315

**Published:** 2026-09-24

**Authors:** Nell Hirt, Enzo Manchon, Ming Wu, Helene Le Buanec, Pauline Bataille, Emmanuelle Bourrat, Jean‐David Bouaziz, Nabila Jabrane‐Ferrat, Reem Al‐Daccak

**Affiliations:** ^1^ Saint Louis Research Institute Saint‐Louis Hospital University of Paris INSERM CNRS, IRSL Paris France; ^2^ Infinity–Institute for Infectious and Inflammatory Diseases University of Toulouse CNRS INSERM Toulouse France; ^3^ AP‐HP Department of Dermatology Saint‐Louis Hospital Paris France

**Keywords:** extravillous trophoblasts, extracellular vesicles, Inflammation, monocytes, recessive dystrophic epidermolysis bullosa

## Abstract

Redirecting immune responses toward tissue repair represents a promising strategy for treating inflammatory and degenerative diseases. Extracellular vesicles (EVs), derived from allogeneic stem cells, have shown potential for modulating proinflammatory immunity. Among these, placental‐derived EVs (PEVs) are emerging as candidates for immune‐mediated inflammatory disorders due to their role in establishing maternal immune tolerance. However, their applicability beyond pregnancy remains limited by key translational barriers, including the lack of a standardized and scalable source system and need to demonstrate immunomodulatory activity in non‐pregnancy allogeneic contexts. In this study, we identify extravillous trophoblasts (EVTs), which play critical role in early placental development, as key source of immunoregulatory PEVs. To overcome donor variability, we employed the HIPEC cell model as established surrogate for primary EVTs and assessed the effects of their derived EVs (EVT‐EVs) on circulating monocytes, pivotal mediators of inflammatory responses. High‐dimensional molecular profiling revealed that EVT‐EVs, similar to EVs derived from first‐trimester primary human trophoblast, carry a distinctive anti‐inflammatory signature, including a unique miRNA repertoire enriched in immunoregulatory pathways. Integrated proteomic and lipidomic analyses demonstrated that EVT‐EVs induce coordinated functional reprograming of monocytes toward an immunomodulatory, inflammation‐resolving phenotype. Notably, EVT‐EVs also modulated the inflammatory bias of monocytes from patients with recessive dystrophic epidermolysis bullosa (RDEB), a genetic disorder characterized by chronic inflammation, thereby promoting restoration of immune balance. Collectively, these findings identify EVT‐EVs as a scalable, off‐the‐shelf platform with high translational potential for the treatment of chronic inflammatory diseases.

## Graphical abstract

First‐trimester trophoblast EVs are enriched in anti‐inflammatory proteins, lipids, and immunoregulatory miRNAs. EVT‐EVs reprogram monocytes toward a pro‐resolving phenotype and restore immune homeostasis in RDEB, supporting their potential as offering a scalable off‐the‐shelf therapy for chronic inflammatory diseases.

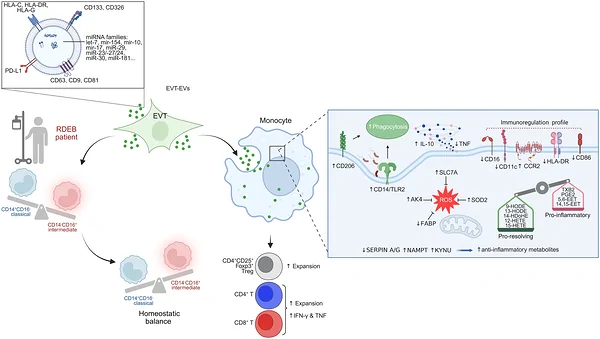

## 1 Introduction

Inflammation, orchestrated by both innate and adaptive immunity, is a fundamental biological response that defends against pathogens and promotes tissue repair, thereby preserving tissue integrity and homeostasis (Netea et al. 2017). Yet, when unresolved, it becomes pathological and contributes to a broad spectrum of chronic immune‐related, metabolic and degenerative disorders. Persistent proinflammatory immunity is also a hallmark of rare genetic disorders, such as recessive dystrophic epidermolysis bullosa (RDEB), a severe inherited skin disorder caused by mutations in the gene encoding type VII collagen. In RDEB, chronic tissue injury drives progressive multi‐organ fibrosis sustained by unresolved inflammation, and no curative treatment is currently available (Bernasconi et al. 2021; Hirt et al. 2025; Popp et al. 2024).

The inflammatory response is coordinated by complex signalling pathways that regulate cytokine and mediator release from tissue‐resident and infiltrating immune cells (Netea et al. 2017). Monocytes are central to this process, acting as sentinels of innate immunity and amplifiers of inflammation when persistently activated (Ginhoux and Jung 2014). In chronic settings, they produce excessive tumour necrosis factor (TNF) and reactive oxygen species (ROS), thereby exacerbating tissue damage (Geng et al. 2016; Kapellos et al. 2019), as we observed in RDEB (Hirt et al. 2025). Strategies that redirect proinflammatory monocytes toward reparative phenotypes therefore, represent a promising therapeutic avenue.

Small extracellular vesicles (EVs) (∼50–150 nm) are nanosized lipid bilayer‐enclosed particles secreted by virtually all cell types that mediate intercellular communication by transferring proteins, lipids, and miRNAs (Mathieu et al. 2019; Witwer and Thery 2019). These vesicles circulate abundantly in biological fluids and play key roles in physiological and immune regulation (Kalluri 2024; Mathieu et al. 2019). Over the past decade, stem cell–derived EVs have emerged as promising modulators of inflammation in degenerative and immune‐mediated disorders (Al‐Daccak and Charron 2018; Hocine et al. 2019; Lima Correa et al. 2021; Madhu et al. 2024; Manchon et al. 2021; Nunes et al. 2025). Despite this promise, clinical translation remains limited by the challenge of establishing safe, standardized and Good Manufacturing Practice (GMP)‐compliant sources of allogeneic EVs (Maumus et al. 2020).

Pregnancy offers a unique paradigm of immune adaptation, often described as “natural transplant”, in which maternal immunity tolerates the semi‐allogeneic fetus without compromising host defense (Jabrane‐Ferrat 2019). This balance is orchestrated by the placenta, a major source of extracellular vesicles during gestation. Placental extracellular vesicles (PEVs) released at the maternal‐fetal interface into the maternal circulation, disseminate systemically and contribute to the modulation of maternal immune responses. Evidence from healthy and pathological pregnancies indicates that PEVs shape monocyte functions, restrain inflammation and promote tolerance (Bai et al. 2022; Wang et al. 2021; Xu et al. 2021). These observations position PEVs as promising immunomodulatory agents; however, their application beyond pregnancy remains limited by the lack of a standardized, scalable sources and evidence of immunomodulatory activity in non‐pregnancy allogeneic contexts.

PEVs originate predominantly from multiple trophoblast cell types, including cytotrophoblasts (CTBs), syncytiotrophoblasts (STBs), and extravillous trophoblasts (EVTs) (Adam et al. 2017; Rosenfeld 2024). Among these, EVTs are immunologically active owing to their invasive phenotype, direct interactions with maternal immune cells, and selective expression of classical and non‐classical HLA class I molecules (HLA‐C, HLA‐E, and HLA‐G) (Tilburgs et al. 2015; Vento‐Tormo et al. 2018). Their EVs production, combined with potent immunomodulatory capacity, identifies EVT‐derived EVs (EVT‐EVs) as potential central regulators of maternal immune adaptation.

To investigate their translational potential, we established a scalable source of EVT‐EVs using the human invasive proliferative extravillous cytotrophoblast (HIPEC) cell line (Pavan et al. 2003), thereby overcoming the variability inherent to primary trophoblasts. Using integrated functional, proteomic, lipidomic, and miRNAomic analyses, we show that EVT‐EVs reprogram monocytes toward an immunomodulatory anti‐inflammatory phenotype. Notably, EVT‐EVs also modulated the pro‐inflammatory bias of monocyte subsets in RDEB, promoting immune homeostasis. These findings provide mechanistic insight into EVT‐EVs biology and support their potential as a scalable and standardized, off‐the‐shelf platform for treatment of chronic inflammatory diseases.

## 2 Materials and Methods

### 2.1 Study Design

To establish a scalable and standardized source of EVT‐EVs, we employed human invasive proliferative extravillous cytotrophoblast (HIPEC) cell line (Pavan et al. 2003) as a consistent alternative to primary trophoblasts, which are subject to inter‐donor variability, and assessed their translational potential by determining their effects on circulating monocytes. To isolate monocytes, we used fresh blood samples from healthy donors and from our small clinical‐biological cohort of adult RDEB patients diagnosed at birth with different COL7A1 pathologic variants and followed at the Dermatology Department—French national reference centre for rare diseases of the skin and mucous membranes of genetic origin (MAGEC)—Saint‐Louis Hospital (Hirt et al. 2025). We applied a combination of flow cytometry and functional assays as well as high‐dimensional proteomic, lipidomic, and miRNAomic analyses to monitor behaviour of EVT‐EVs‐educated monocytes.

### 2.2 Cell Culture

HIPEC cells, referred to as extravillous trophoblasts (EVTs) in this study, were cultured in Dulbecco's Modified Eagle's Medium (DMEM) supplemented with 10% of Fetal Bovine Serum (FBS), 1% of Penicillin/Streptomycin solution and 1% of L‐Glutamine (Gibco). Cultured cells were routinely tested for Mycoplasma using the MycoBlue Mycoplasma Detector kit (Vazyme).

### 2.3 Isolation of EVT‐EVs

Culture medium supplemented with EVs‐depleted serum was obtained by overnight ultracentrifugation at 100.000 g (Optima XPN‐80, Beckman Coulter) of DMEM supplemented with 30% FBS, 1% of Penicillin/Streptomycin solution and 1% of L‐Glutamine solution. After ultracentrifugation, EVs‐depleted supernatant was carefully pipetted from the top, leaving 5 mL in the bottom of each tube to avoid disturbing bottom layers or the pellet. Supernatants were filtered using Stericup Quick Release‐GP sterile vacuum filtration system (0.22µm pore; Sigma‐Aldrich), and additional DMEM medium supplemented 1% of Penicillin/Streptomycin solution and 1% of L‐Glutamine solution were added to obtain DMEM culture medium supplemented with 10% EVs‐depleted FBS.

EVT‐EVs were isolated by differential ultracentrifugation using standardized protocols (Hocine et al. 2019) and following latest International Society for Extracellular vesicles (ISEV) guidelines (Welsh et al. 2024). Briefly, EVTs were cultured in 10% EVs‐depleted medium for 48 h. Conditioned medium was then harvested and filtered using Stericup Quick Release‐GP sterile vacuum filtration system (0.22µm pore; Sigma‐Aldrich). Supernatants were processed by three successive ultracentrifugation (Optima XPN‐80, Beckman Coulter) steps at 100.000 g and 4°C to isolate EVT‐EVs fractions. All pellets were washed in sterile PBS and re‐centrifuged at 100.000 g and 4°C before being resuspended in sterile PBS and stored at ‐80°C.

### 2.4 Nanoparticle Tracking Analysis of EVT‐EVs

Average size distribution and particle concentration analysis of EVT‐EVs samples were performed by Nanoparticles Tracking Analysis (NTA) according to the manufacturer's software manual (Nanosight LM10 User Manual). Briefly, camera level was increased until all particles were distinctly visible not exceeding a particle signal saturation over 20%. Autofocus was adjusted so that indistinct particles were avoided. For each sample/measurement, five 1‐min videos were captured under specific conditions (cell temperature: 25°C; Syringe speed: 40 µL/s). After capture, the videos were analysed by the in‐build Nanosight Software NTA 3.1 Build 3.1.46 with a detection threshold of 5 to obtain particle concentration (particle/mL). PBS and supernatant (after ultracentrifugation) controls were also analyzed by NTA. All isolated EVT‐EVs samples were diluted in PBS at a dilution of 1/1000 for measurements.

### 2.5 Immunoblotting

Cells or EVT‐EVs were lysed with 1X RIPA buffer (Thermofisher scientific) supplemented with proteinase inhibitor for 1 h at 4°C. Proteins were separated on 10% SDS‐PAGE under reducing or non‐reducing conditions (depending on the antibody) and transferred onto a nitrocellulose membrane using InvitrogeniBlot2 system. Membranes were blocked for 1 h at room temperature in 1X TBS with 0.1% Tween (TBST; Thermo Fisher Scientific) containing 2% BSA, followed by overnight incubation at 4°C with primary antibodies (anti‐CD63, ‐PAP, ‐TSG101, ‐ADAM10, and ‐Syntenin‐1; 1:1000 dilution in TBST/2% BSA). After washing in TBST, membranes were incubated for 1 h with HRP‐conjugated secondary antibody (Bio‐Rad; 1:3000 in TBST/2% BSA), washed again, and developed using SuperSignal West Pico PLUS Chemiluminescent Substrate (Thermo Fisher Scientific) for 5 min. Chemiluminescence was detected with ImageQuant LAS 4000/4010 (GE Healthcare), and immunoblots were analyzed using ImageJ software (v1.51).

### 2.6 Bead‐Based Multiplex Flow Cytometry Assay

EVT‐EVs were characterized using human MACSPlex Exosome Kit (Miltenyi Biotec) according to the manufacturer's instructions. Briefly, EVT‐EVs samples were diluted in MACSPlex buffer (MPB) to a final volume of 120 µL and incubated with 15 µL of MACSPlex EV capture beads, each conjugated to one of the 39 exosomal marker specific antibodies. A detection reagent containing APC‐conjugated anti‐CD9, anti‐CD63, and anti‐CD81 antibodies (15 µL) was then added, and samples were incubated for 1 h at room temperature in the dark on an orbital shaker at 450 rpm. During acquisition, median fluorescence intensity (MFI) for all 39 exosomal markers were background‐corrected and gated based on bead‐specific fluorescence according to kit specification. Flow cytometry data were acquired using a BD FACSCanto (BD biosciences) and analyzed with FlowJo software (v10, FlowJo LLC).

### 2.7 Monocytes Isolation

CD14^+^CD16^−^ monocytes were isolated from peripheral blood mononuclear cells (PBMCs) by negative magnetic selection using the human classical monocyte isolation kit, human (Miltenyi Biotec), following manufacturer's instructions. For experiments involving RDEB patients, CD14^+^ monocytes were directly isolated from whole blood samples of patients and healthy controls using the StraightFrom Whole Blood CD14 MicroBeads kit (Miltenyi Biotec). In all experiments, isolated cells were resuspended in RPMI‐1640 medium supplemented with 10% human serum, 1% Penicillin‐Streptomycin, and 1% L‐glutamine (complete medium) and used as specified in the respective assays.

### 2.8 EVT‐EVs Cellular Internalization

EVT‐EVs were labelled with Carboxyfluorescein N‐succinimidyl ester (CFSE) by incubation in PBS for 45 min at 37°C, followed by overnight incubation at 4°C. Excess dye was removed by washing twice with PBS using ultracentrifugation at 100,000 × g at 4°C. for 2 h. Average size distribution and particle concentration analysis of CFSE‐labelled EVT‐EVs samples were determined by NTA as described above. Uptake of CFSE‐labelled EVT‐EVs by monocyte was assessed using confocal microscopy and flow cytometry. For confocal microscopy, monocytes (1 × 10^5^ cells) were seeded onto Ibidi glass‐bottom chamber slides (Ibidi, Germany) and allowed to adhere for 24 h at 37°C in 5% CO_2_. Cells were washed with PBS and incubated with CFSE‐labelled EVT‐EVs for 4 h. Cells were then fixed with 4% paraformaldehyde (PFA), incubated with Fc receptor‐blocking solution, and stained with PE‐conjugated anti‐HLA‐I antibody for 1 h at 4°C. Slides were mounted using Ibidi mounting medium with DAPI, and images were acquired at ×200 and ×400 magnification using LSM 780 MP microscope. For flow cytometry analysis, monocytes (1 × 10^5^ cells) were incubated with CFSE‐labelled EVT‐EVs in 24‐well plates for 30 min, 1, 2, 4, 6, or 8 h. Cells were harvested and analyzed using a BD Fortessa flow cytometer, and data were processed using FlowJo software (v10.8.1).

### 2.9 Immunophenotyping and Flow Cytometry

Monocytes were seeded in 24‐well plates at 4×10^5^ cells/mL in complete medium (RPMI‐1640 supplemented with 10% human serum, 1% Penicillin‐Streptomycin, and 1% L‐glutamine) and cultured for 3 days at 37°C in 5% CO2, in the presence or absence of 0.5 × 10^11^ EVT‐EVs/mL. Cells were then harvested and their phenotype assessed using specific antibodies (Table S1) and flow cytometry. Briefly, harvested cells were incubated with Fc receptor blocking reagent for 20 min at 4°C to reduce nonspecific binding, followed by staining with specific antibodies for 45 min at 4°C. For intracellular markers, cells were fixed, permeabilized and stained using the Foxp3/Transcription Factor Staining Buffer Set (eBioscience) according to manufacturer's instructions. Intracellular antibodies were used at 1:50 dilution, and LIVE/DEAD solution at 1/500 dilution. Data acquisition was performed on a BD Fortessa flow cytometer, and analyses were carried out with FlowJo software (10.8.1). Unsupervised clustering was performed based on marker expression values using FlowSOM or PhenoGraph algorithms implemented on the OMIQ platform (www.omiq.ai).

### 2.10 Phagocytosis Assay

Isolated monocytes were seeded in 24‐well plates at 4 × 10^5^ cells/mL in complete medium and cultured for 3 days at 37°C in 5% CO2, in the presence or absence of 0.5 × 10^11^ EVT‐EVs/mL. Lipopolysaccharide (LPS, 50 ng/mL) was added during the final 16 h of culture. Cells were then harvested and phagocytic activity was assessed by measuring cellular uptake of zymosan A bioparticles. Briefly, cells were incubated with 0.5 mg/mL Red pHrodo Zymosan A bioparticles in complete medium for 90 min at 37°C. To stop phagocytosis and remove the excess of particles, cells were washed in cold 1X PBS. Uptake was quantified by flow cytometry, with phagocytic cells identified as pHrodo‐positive and 7‐AAD‐negative.

### 2.11 Mitochondrial Mass and Reactive Oxygen Species (ROS) Assessment

Isolated monocytes were seeded in 24‐well plates at 4 × 10^5^ cells/mL in complete medium and cultured for 3 days at 37°C in 5% CO2, in the presence or absence of 0.5 × 10^11^ EVT‐EVs/mL. LPS (50 ng/mL) was added during the final 16 h of culture. Cells were then assessed for mitochondrial mass and reactive oxygen species (ROS) production. Mitochondrial mass was measured using MitoTracker Green FM (Invitrogen, M7514) following the manufacturer's instructions. Briefly, after 3 days, monocytes were washed and incubated with 100 nM MitoTracker Green FM for 30 min at 37°C, washed twice with cold 1X PBS, acquired with BD FACSCanto (BD biosciences), and analyzed using FlowJo (10.8.1) software. Intracellular ROS levels were evaluated using the DCFDA/H2DCFDA Cellular ROS Assay Kit (Abcam) as per manufacturer's instructions. Briefly, after 3 days, monocytes were washed and incubated with 25 µM DCFDA for 30 min at 37°C, then directly acquired on BD FACSCanto (BD biosciences) and analyzed using FlowJo (10.8.1) software.

### 2.12 Cytokines Profiling

Monocytes were seeded in 24‐well plates at 4 × 10^5^ cells/mL in complete medium and cultured for a total of 3 days at 37°C in 5% CO2, in the presence or absence of 0.5 × 10^11^ EVT‐EVs/mL. LPS (50 ng/mL) was added during the final 16 h of culture. Supernatants were collected, and cytokine levels were quantified using the MACSPlex Cytotoxic Basic Kit (Miltenyi Biotec) according to the manufacturer's instructions. Data acquisition was performed on a BD FACSCanto (BD biosciences) and analyzed using FlowJo (10.8.1) software.

### 2.13 Immunomodulation Assays

The effects of EVT‐EVs on adaptive immune responses were assessed in allogenic co‐culture settings. Briefly, carboxyfluorescein succinimidyl ester (CFSE, Fisher Scientific)‐labelled PBMCs (responders) were co‐cultured with HLA‐mismatched monocytes at a 5:1 ratio for 5 days, with or without EVT‐EVs. Alternatively, HLA‐mismatched CFSE‐labelled PBMCs were stimulated with ImmunoCult Human CD3/CD28/CD2 T Cell Activator (StemCell Technologies, 25µL/million cells) for 3 days in the presence or absence of monocytes (5:1 ratio) with or without EVT‐EVs. At the end of the cultures, cells were stained with anti‐CD3, ‐CD8, ‐CD4, ‐CD25, ‐FoxP3, anti‐TNF and anti‐IFNγ antibodies (Table 1). T cell subset frequencies, proliferation and cytokine production were determined by flow cytometry and analyzed using FlowJo software (10.8.1).

**TABLE 1:** Antibodies used in this study.

Antibody	Clone	Source	Cat # identifier
CD14‐PE	REA599	Miltenyi	130‐110‐577
CD16‐BV711	3G8	BD Bioscience	563127
CD11c‐FITC	B‐Iy6	BD Bioscience	561355
CCR2‐APC	K036C2	Biolegend	357208
HLA‐DR‐BV421	G46‐6	BD Bioscience	562804
CD86‐PECF594	2331	BD Bioscience	562390
CD25‐PE	M‐A251	BD Bioscience	555432
FoxP3‐APC	236A/E7	Invitrogen	17‐4777‐42
CD4‐APCH7	RPA‐T8	BD Bioscience	560158
CD8‐BV395	RPA‐T8	BD Bioscience	563795
CD163‐BV421	GHI/61	BD Bioscience	562643
CD36‐BV395	CB38	BD Bioscience	565422
CD64‐PECY7	RE978	Miltenyi	130‐116‐301
CD206‐APC	19.2	BD Bioscience	561763
IFNg‐PECY7	B27	BD Bioscience	557643
TNF‐PE	MAb11	BD Bioscience	559321

### 2.14 MiRNA Sequencing

Exosomal RNA was isolated using QIAzol Lysis Reagent (QIAGEN) supplemented with an RNA spike‐in template mixture, following manufacturer instructions. After a 5 min incubation at room temperature, chloroform was added, samples were vortexed, incubated for 2–3 min, and centrifuged at 12,000xg for 15 min at 4°C. The aqueous phase was mixed with 1.5 volumes of 100% ethanol and applied in 700 μ aliquots to RNeasy MinElute spin columns (QIAGEN). Columns were centrifuged at 8000xg for 15 s, sequentially washed with Buffer RWT, Buffer RPE, and 80% ethanol, and eluted in 40 µL RNase‐free water following a 1‐min incubation and centrifugation at 15,000xg. Reverse transcription was performed on 19 µL RNA using the miRCURY LNA RT Kit (QIAGEN) in 95 µL reactions. cDNA was diluted 1:50 and amplified in 10 µL reactions using the miRCURY LNA miRNA PCR System (Human Panels I+II) and SYBR Green master mix. Reactions were run on a LightCycler 480 System (Roche) in 384‐well format, including no‐template RT controls. Cq values were determined using the second derivative method (Roche LC software). Specificity was confirmed via melting curves, and amplification efficiency was evaluated using a LinRegPCR‐like algorithm. Only assays showing a single melting peak at the expected Tm, Cq < 37, and at least 5 Cq lower than controls were retained. Cq values were normalized to the global mean or to a subset of miRNAs consistently detected across all samples.

### 2.15 Proteomic Profiling

Cell lysates were solubilized in 50 mM ammonium bicarbonate buffer (pH 8.0), and protein concentrations were determined using a BCA assay (Sigma‐Aldrich), then adjusted to 1 mg/mL with additional buffer. Proteins were processed using the ProteinWorks eXpress kit (Waters Corporation) according to the manufacturer's instructions. Briefly, 40 µL of each sample was incubated at 80°C for 10 min in digestion buffer containing RapidGest detergent solution (7 mg/mL). Proteins were reduced with 70 mM dithiothreitol for 20 min at 60°C and alkylated with 140 mM iodoacetamide for 30 min at room temperature in the dark. Samples were digested overnight at 37°C with trypsin at enzyme‐to‐protein ratio of 1:25. Digestion was quenched with 20% trifluoroacetic acid and incubated for 15 min at 45°C. Precipitates were removed by centrifugation at 10,000xg for 15 min at 10°C. Peptide‐containing supernatants were desalted using 30 mg Oasis HLB cartridges (Waters Corporation), preconditioned with 1 mL of 100% methanol and equilibrated with 1 mL of water. Samples were loaded onto the cartridges, washed with 1 mL of 5% methanol, and eluted with 500 µL of 80% methanol. Eluates were dried under nitrogen at 45°C, reconstituted in 100 µL of 5% acetonitrile containing 0.1% formic acid, and 1 µL was injected for nano‐liquid chromatography‐tandem mass spectrometry (nanoLC‐MS/MS) analysis. Peptide separation was performed using an Orbitrap Exploris 480 mass spectrometer coupled to a Vanquish Neo nanoLC (ThermoFisher Scientific). Samples were loaded onto a PepMap C18 precolumn (300 µm × 5 mm) and separated on a 75 µm × 500 mm EASY‐Spray PepMap Neo analytical column using a 60‐min gradient of mobile phase B (80% acetonitrile with 0.1% formic acid) in mobile phase A (0.1% formic acid in water) at 300 nL/min. Data were acquired in data‐dependent acquisition (DDA) mode using Xcalibur software (v4.2), with MS1 and MS2 resolutions set to 120,000 and 15,000, respectively.

Peptides and proteins were identified and quantified using Proteome Discoverer (version 2.5.0400, ThermoFisher Scientific) against the *Homo Sapiens* Uniprot reference proteome and a common contaminant database. Trypsin was set as the cleavage enzyme, allowing up to 2 missed cleavages. Search parameters included fixed carbamidomethyl (+57.0214 Da) and variable modifications for N‐terminal acetyl (+42.0105 Da), and methionine oxidation (+15.9949 Da). A minimum peptide length of six amino acids with at least one unique peptide per protein were required. The false discovery rates (FDR) were set to 1% at peptide‐spectrum match, protein, and modification site levels. Label‐free MS1‐based quantification was performed within Proteome Discoverer.

### 2.16 Global Proteomic Data Analysis

Raw MS/MS data were processed using MaxQuant (version 1.5.1.2) for protein identification and label‐free quantification (Cox and Mann 2008). MS/MS spectra were searched against the human proteome database, excluding non‐host proteins when relevant. Pre‐processing was performed using the data Process function from the MSstats package (Choi et al. 2014), with noise filtering enabled (feature Subset = “highQuality” and remove_uninformative_feature_outlier = TRUE). Protein intensities were log_2_‐transformed and *Z*‐score normalized by autoscaling. Differential protein abundance was assessed using the edgeR package (Robinson et al. 2010), considering proteins with adjusted *p* < 0.05 and absolute fold change > 1.5 as significant. Principal component analysis (PCA) was performed using the PCA2D package, and hierarchical clustering of the top 100 differentially expressed proteins (50 upregulated and 50 downregulated) was visualized using the Complex Heatmap package (Gu et al. 2016). Functional enrichment analysis was conducted via Ingenuity Pathway Analysis (IPA), and visualization was created in R (R Core Team. R: A language and environment for statistical computing. R Foundation for Statistical Computing; 2024. Available from (https://www.R‐project.org/) using ggplot2 (Wickham H. ggplot2: Elegant Graphics for Data Analysis (https://ggplot2.tidyverse.org), and circlize (Gu et al. 2014).

### 2.17 Lipid Extraction and Quantitative Analysis by LC‐MS/MS

Monocytes cultured or not with EVT‐EVs for three days were harvested, washed and snap‐frozen in liquid nitrogen and stored until lipid extraction and analysis as we described (Chen et al. 2020). Briefly, 1 × 10^6^ cells/sample were used for protein quantification using the Bio‐Rad Protein Assay. Internal standards (IS), including deuterium‐labelled bioactive lipids (LxA4‐d5, LtB4‐d4, 5‐HETE‐d8; 400 ng/mL, Sigma‐Aldrich and Cayman Chemical), were added to each sample. Targeted quantitative LC–MS/MS was employed to simultaneously separate the 36 lipids of interest, including bioactive eicosanoids and related lipid mediators, and the three deuterium‐labelled internal standards. Lipid extraction was initiated by adding 300 µL of cold methanol and 5 µL of IS to each sample, followed by centrifugation. After centrifugation, supernatants were subjected to solid‐phase extraction (SPE), and lipid mediators were subsequently eluted and analyzed via liquid chromatography‐tandem mass spectrometry (LC‐MS/MS). Data acquisition was performed in multiple reaction monitoring (MRM) mode using optimized instrument settings, including ion optics and collision energies. Peak detection, integration, and quantification of bioactive eicosanoids and related lipid mediators were conducted using MassHunter Quantitative Analysis software (Agilent Technologies).

### 2.18 Statistical Analysis

Statistical analysis was performed using GraphPad Prism 9.5.0 (GraphPad Software). Data on the graphs represent the mean ± SEM from each group and comparisons were performed using paired or unpaired t‐test assuming Gaussian distribution with parametric test if normality is obtained with Anderson‐Darling (A2*)/D'Agostino‐Pearson omnibus (K2)/Shapiro‐Wilk (W)/Kolmogorov‐Smirnov (distance), or non‐parametric test with Mann Whitney test if normality is not obtained. *P*‐values < 0.05 were considered statistically significant. Based on the proteomic and lipidomic analysis, clusters obtained from monocytes treated or not with EVT‐EVs were subjected to Principle Component analysis (PCA).

## 3 Results

### 3.1 EVTs Release Immunoregulatory Extracellular Vesicles with Anti‐Inflammatory Potential

The human invasive proliferative extravillous cytotrophoblast (HIPEC) cell line is a validated model of first‐trimester extravillous trophoblasts (EVTs), offering a stable and homogeneous epithelial population that recapitulates key phenotypic and functional features of primary EVTs (Pavan et al. 2003). HIPEC cells express the typical epithelial trophoblast marker cytokeratin‐7 (CK7) and display EVT‐typical immune profile, including classical HLA‐C, and non‐classical HLA‐G and HLA‐E class I molecules (Pavan et al. 2003). To establish a scalable and standardized source of EVT‐derived extracellular vesicles, we employed HIPEC as a consistent alternative to primary EVTs, thereby circumventing the variability between donors.

**FIGURE 1:**
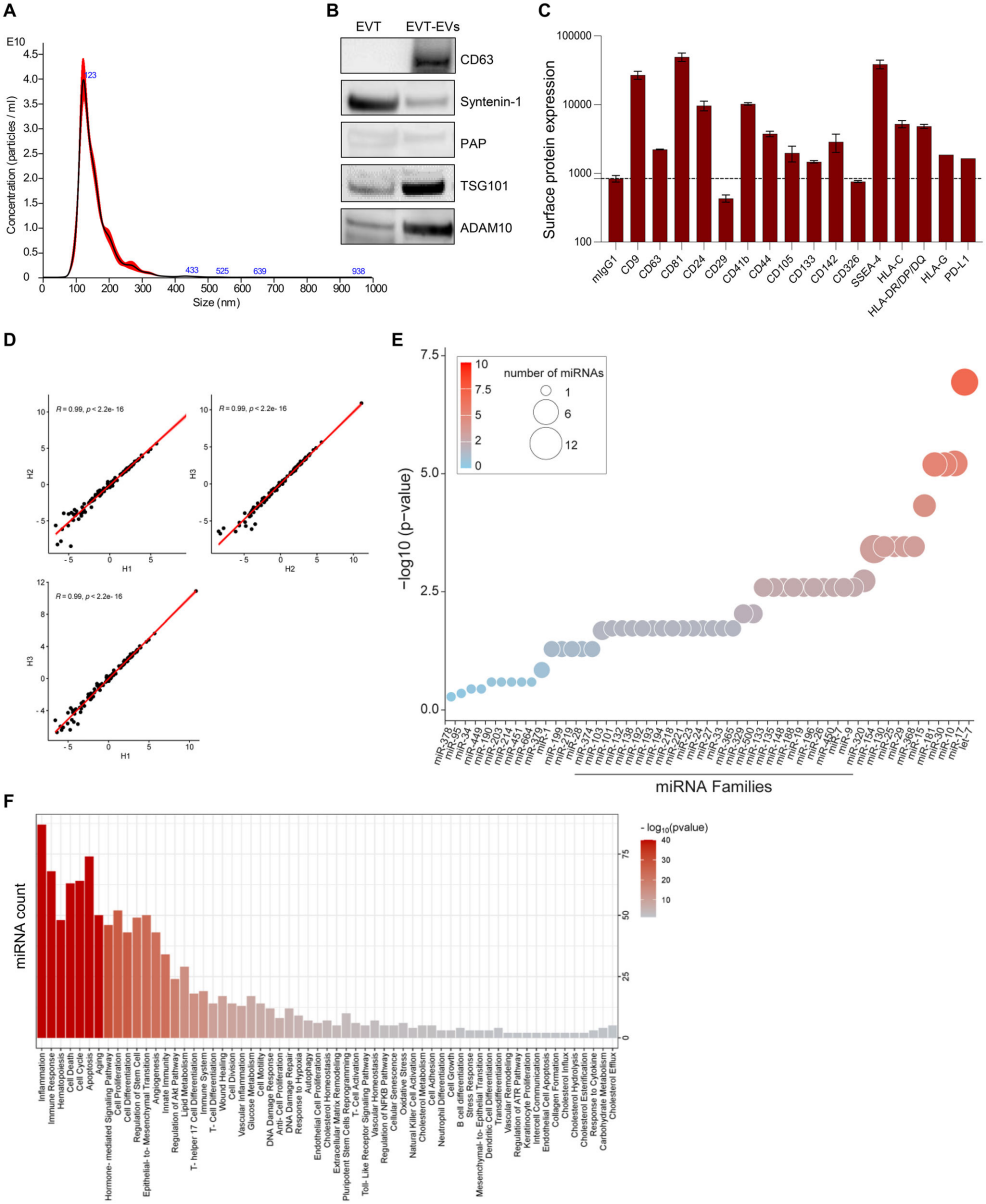
EVT‐derived extracellular vesicles (EVT‐EVs) display immunomodulatory signature. (A) Nanoparticle tracking analysis (NTA) showing size distribution of EVT‐EVs. (B) Western blot of CD63, syntenin‐1, PAP, TSG101 and ADAM10 in EVT‐EVs and the parental HIPEC extravillous trophoblast cell line. (C) Surface protein expression profile presented as log_10_‐transformed geometric mean intensity. (D) Pearson and Spearman correlation of three independent EVT‐EVs preparations. (E) Bubble plot of enriched miRNA families in EVT‐EVs. Bubble color (light blue to red) reflects statistical significance (−log_10_ *p* value); bubble size indicates the number of miRNAs per family. (F) Functional enrichment of EVT‐EV miRNAs shown as a bar plot. The *x*‐axis denotes cellular or molecular functions; the *y*‐axis, the number of associated miRNAs. Color intensity indicates statistical significance (−log_10_ *p* value; deeper red = greater significance).

Extracellular Vesicles isolated from HIPEC culture supernatants (EVT‐EVs) were enriched in small vesicles within the expected size range of 50–150 nm (Figure 1A). EVT‐EVs expressed placental alkaline phosphatase (PAP) and the universal exosome marker syntenin‐1, together with components of the ESCRT (endosomal‐sorting complexes required for transport) machinery, including TSG101, and ADAM10. Canonical tetraspanins—CD9, CD63, and CD81—were also detected (Figures 1B,C). At their surface, EVT‐EVs display markers characteristic of first‐trimester trophoblast identity and stemness (CD326, CD41b, CD105, CD133, and SSEA‐4), as well as immunoregulatory molecules including HLA‐C, HLA‐G, HLA‐II antigens, and PD‐L1 (Figure 1C). Notably, EVT‐EVs displayed comparable size distribution, expression of canonical EV and surface marker profiles as EVs derived from first‐trimester primary human trophoblast (PHT‐EVs) (Figures S1A–C).

Given the established role of EV‐associated microRNAs (miRNAs) in immunoregulation (Bartel 2009; Ding et al. 2025), we next profiled the miRNA cargo of EVT‐EVs. miRNome analysis across three independent preparations showed high reproducibility (Pearson *r* = 0.99, *P* < 0.05; Figure 1D). Using TAM 2.0 enrichment, we identified 35 miRNA families enriched in EVT‐EVs, including the let‐7, miR‐154, miR‐17, miR‐10, miR‐30, and miR‐181 families—each implicated in the regulation of inflammation, immunity, and metabolism (Figure 1E). Similar miRNA immunomodulatory signature was observed with PHT‐EVs (Figure S1D). Pathway enrichment analysis further indicated that these miRNAs target innate immune responses, inflammatory signalling, and metabolic pathways (Figure 1F). Together, these data suggest that EVT‐EVs similar to PHT‐EVs carry a miRNA cargo with significant immunomodulatory and anti‐inflammatory potential.

### 3.2 EVT‐EVs Drive Anti‐Inflammatory Proteome Remodelling in Monocytes

**FIGURE 2:**
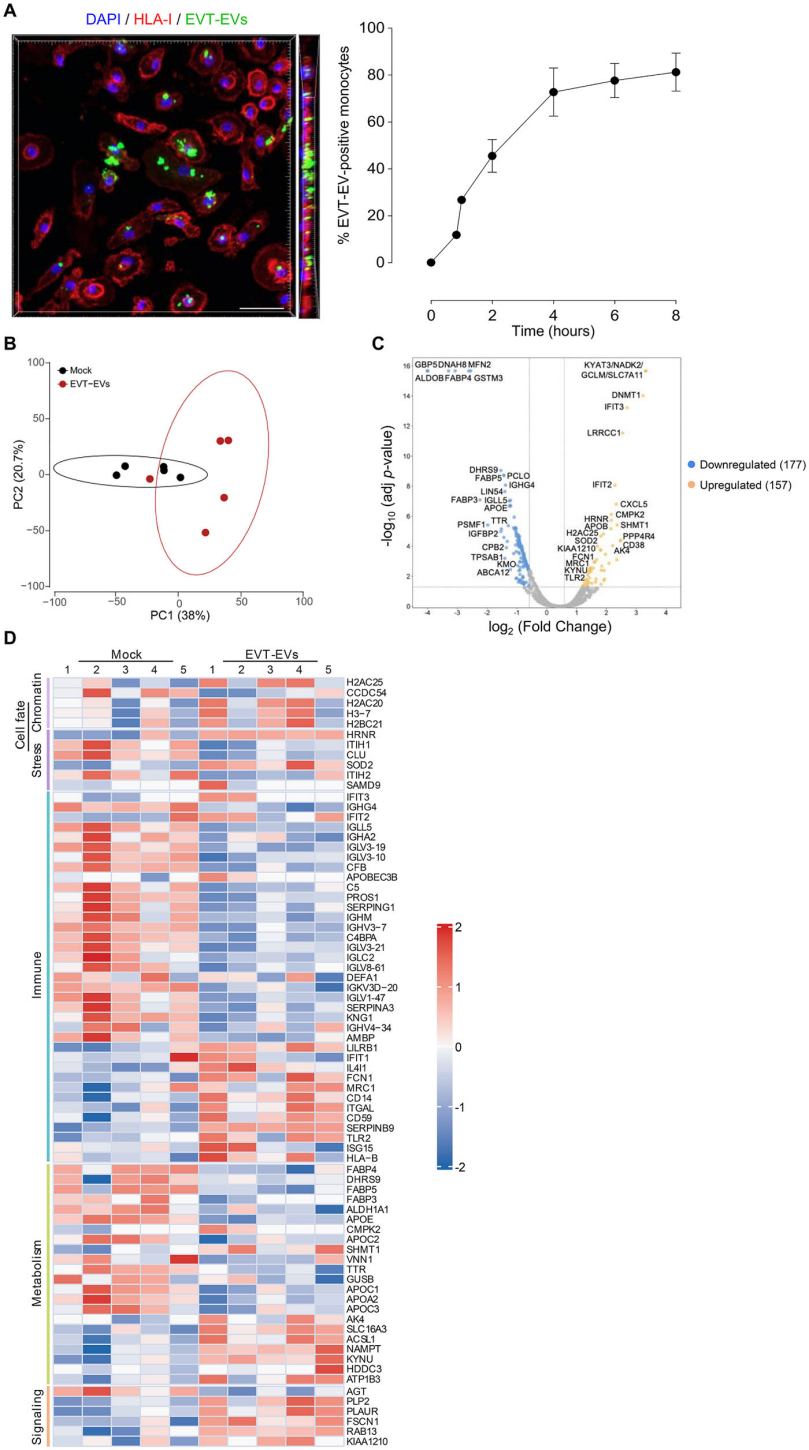
EVT‐EVs induce anti‐inflammatory reprogramming of the monocyte proteome. (A) Representative confocal image of CFSE‐labelled EVT‐EVs uptake of (green) by peripheral blood monocytes. Monocyte membranes are stained for HLA‐I (red) and nuclei with DAPI (blue). Right, proportion of CFSE^+^ monocytes at indicated time points (mean ± SEM, *n* = 3). (B) PCA of proteomic profiles from EVT‐EVs‐ and mock‐treated monocytes. (C) Volcano plot showing differentially expressed proteins (DEPs) between groups. Proteins with *p* < 0.05 and fold change > 1.5 are considered significant. (D) Heatmap of significantly regulated proteins. Columns represent individual samples; rows, proteins. *Z*‐score–normalized expression is shown (red, upregulation; blue, downregulation). Side Bar colors distinguishing functional categories.

To assess the functional relevance of the miRNA cargo within EVT‐EVs, we investigated their interaction with innate immune cells, specifically CD14^+^ monocytes, which play a key role in early immune surveillance and tissue homeostasis. EVT‐EVs were labelled with CFSE, and analysis of their average size distribution (Figure S2A) confirmed the purity of the EV population and excluded the presence of residual free‐dye. CD14^+^ monocytes were isolated from peripheral blood mononuclear cells (PBMCs) of healthy donors and incubated with CFSE‐labelled EVT‐EVs. Confocal imaging and time‐course uptake assays showed rapid internalization by monocytes, with detectable uptake at 60 min and saturation by 8  h (Figure 2A). These findings demonstrate that EVT‐EVs effectively engage these innate immune cells, supporting their potential as immunomodulatory agents.

To evaluate the downstream consequences of EVT‐EVs education, we performed proteomic profiling of CD14^+^ monocytes following a three‐day treatment. This revealed widespread alterations in the monocyte proteome (Figure 2B–D). Principal component analysis (PCA) demonstrated clear segregation of EVT‐EV‐treated and untreated monocytes, indicating a global shift in protein expression (Figure 2B). Differential expression analysis identified 157 proteins significantly upregulated and 177 downregulated in EVT‐EV‐educated monocytes (Figure 2C). Hierarchical clustering of the top differentially expressed proteins showed robust separation between treated and control groups, consistent with reproducible reprogramming of monocyte identity toward a metabolically adaptive and immunoregulatory phenotype (Figure 2D).

Upregulation of core histones H2AC25, H2AC20, H2BC21, and H3 suggests extensive chromatin remodelling, reflecting increased nucleosome assembly and epigenetic plasticity that may underlie shifts in gene expression driving immunomodulation and metabolic adaptations. Concurrent upregulation of superoxide dismutase 2 (SOD2) indicates enhanced mitochondrial antioxidant capacity and resilience to oxidative stress. Downregulation of ITIH1, ITIH2, and CLU further reflects reprogramming of monocyte metabolic pathways toward resolving of pro‐inflammatory acute‐phase mediators. Together, these changes highlight a coordinated program in which chromatin dynamics, stress response, and metabolic rewiring converge to shape monocyte fate toward a regulatory, homeostatic phenotype.

EVT‐EVs‐treated monocytes also exhibited enhanced immune recognition and resolution capacity, with upregulation of CD14, mannose receptor MRC1 (CD206), ficolin‐1 (FCN1), and toll‐like receptor 2 (TLR2), indicative of pathogen‐sensing and immune priming phenotype (Kawai and Akira 2010; Netea et al. 2016). Simultaneously, induction of interferon‐stimulated genes (ISG15, IFIT1, IFIT2, and IFIT3) reflects activation of type I interferon signalling and antiviral readiness frequently observed in regulatory or adaptation‐associated monocyte programs and do not inherently indicate a pro‐inflammatory phenotype (Ivashkiv and Donlin 2014; McNab et al. 2015). Notably, TLR2 upregulation was also observed in monocytes exposed to EVs derived from PHT‐EVs, indicating that this response is unlikely to be an artefact of SV40‐mediated immortalization of the EVT cell line (Figure S2B). The coordinated downregulation of SERPINA3 and SERPING1, together with upregulation of SERPINB9 and CD59, supports a shift from pro‐inflammatory acute‐phase responses toward cell‐intrinsic survival and immune‐regulatory functions, consistent with enhanced resistance to cytotoxic stress.

Metabolic and signaling pathways were also reprogrammed: lipid‐handling proteins (FABP3/4/5, APOs) were downregulated, while enzymes supporting NAD^+^ metabolism, oxidative energy pathways, and immunoregulatory metabolite production (KYNU, NAMPT, ATP1B3, SLC16A1, ACSL1) were upregulated. Concurrently, proteins involved in vesicle trafficking, adhesion, and cytoskeletal dynamics (PLN2, PLAUR, RAB13, FSCN1) were elevated, whereas AGT was downregulated. Collectively, these changes indicate a metabolically flexible, stress‐adapted, and tissue‐repair–oriented monocyte phenotype.

Overall, these findings demonstrate that EVT‐EVs reprogram the monocyte proteome toward a homeostatic, metabolically active, and less inflammatory state, characterized by chromatin remodelling, oxidative stress resilience, phagocytic competence, antiviral preparedness, and reduced inflammasome activation.

### 3.3 EVT‐EVs Activate Immunoregulatory and Metabolic Programs in Monocytes

**FIGURE 3:**
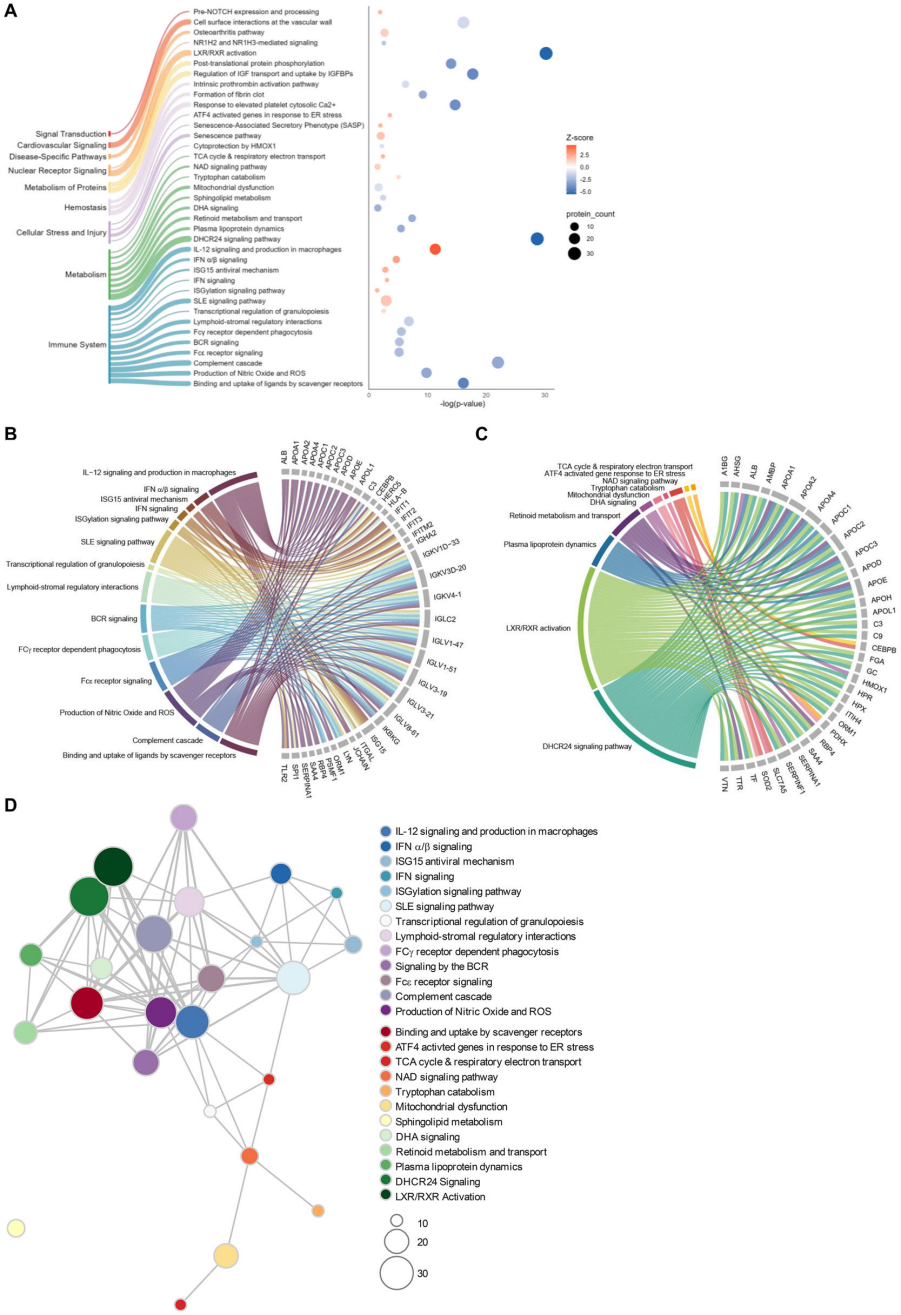
Pathway‐level analysis of the monocyte proteomic response to EVT‐EVs. (A) Enrichment analysis of canonical pathways predicted by Ingenuity Pathway Analysis (IPA). Left, Sankey plot showing significantly enriched pathways grouped by biological function (e.g., immune response, metabolism). Each ribbon represents an individual pathway, with colors distinguishing functional categories. Right, bubble plot visualizing pathway activation status. Bubble color indicates predicted *z*‐score (red, activated; blue, inhibited); bubble size reflects the number of overlapping proteins; the *x*‐axis shows statistical significance (−log_10_ *p* value). Only significantly enriched pathways are shown. (B, C) Chord diagrams of enriched immune‐related (B) and metabolism‐related (C) pathways and their contributing proteins. Ribbons connect proteins to multiple pathways, highlighting shared molecular interactions. (D) Pathway interaction network. Nodes represent significantly enriched immune and metabolic pathways; node size corresponds to the number of associated proteins; color denotes functional category; and edges indicate co‐enrichment or functional similarity.

To gain insight into the biological pathways underpinning EVT‐EVs–induced proteomic reprogramming, we conducted enrichment analysis using Ingenuity Pathway Analysis (IPA). The most significantly enriched pathways, ranked by –log(*p*‐value), are presented in a Sankey plot and encompass processes related to immune regulation, metabolism, and stress response (Figure 3A, left). Immune signaling cascades, such as interferon signaling, TREM1 signaling, and pattern recognition receptor pathways were prominently enriched, consistent with an antiviral, homeostatic monocyte phenotype. Concurrently, mitochondrial metabolic pathways including oxidative phosphorylation and the tricarboxylic acid (TCA) cycle were increased indicating a shift towards oxidative metabolism. A bubble plot integrating predicted pathway enrichment states with associated protein counts further illustrated differential modulation of key cellular programs (Figure 3A, right). Pathways linked to pro‐inflammatory and cytotoxic responses such as IL12 signaling and oxidative stress responses, were predicted to be downregulated. In contrast, stress‐adaptive and immunomodulatory pathways were upregulated, notably the ATF4‐mediated endoplasmic reticulum (ER) stress response, which is associated with anti‐inflammatory macrophage polarization and immune homeostasis. These results point to increased cellular resilience and metabolic adaptability of EVT‐EVs‐educated monocytes.

Contextualization of the biological networks underlying EVT‐EVs‐induced monocyte programming is further illustrated in chord diagrams (Figure 3B,C), showing distinct clusters of protein‐pathway associations enriched in either immune or metabolic functions. To further investigate the functional relationships between EVT‐EVs–regulated pathways, we constructed a pathway interaction network based on co‐enrichment and shared molecular components (Figure 3D). Strikingly, immune‐related pathways, including IL12 signaling, IFN responses, and BCR/Fcγ receptor signaling, formed a densely interconnected module, reflecting coordinated enhancement of innate immune surveillance and antigen recognition. Adjacent to this immune hub, metabolic pathways such as the tricarboxylic acid TCA cycle, lipid metabolism (e.g., LXR/RXR activation and docosahexaenoic acid (DHA) signaling), and ATF4‐mediated stress responses were also tightly clustered, suggesting extensive crosstalk between immunoregulatory and metabolic adaptation programs. More peripheral nodes, such as tryptophan catabolism and retinoid metabolism, further indicate targeted immunometabolic rewiring.

Collectively, these findings support a model in which EVT‐EVs induce selective innate immune reprogramming in monocytes with reduced representation of classical inflammatory activation programs alongside enrichment of regulatory and metabolic, and adaptation‐related processes.

### 3.4 Phenotypic Remodelling of Classical Monocytes by EVT‐EVs

**FIGURE 4:**
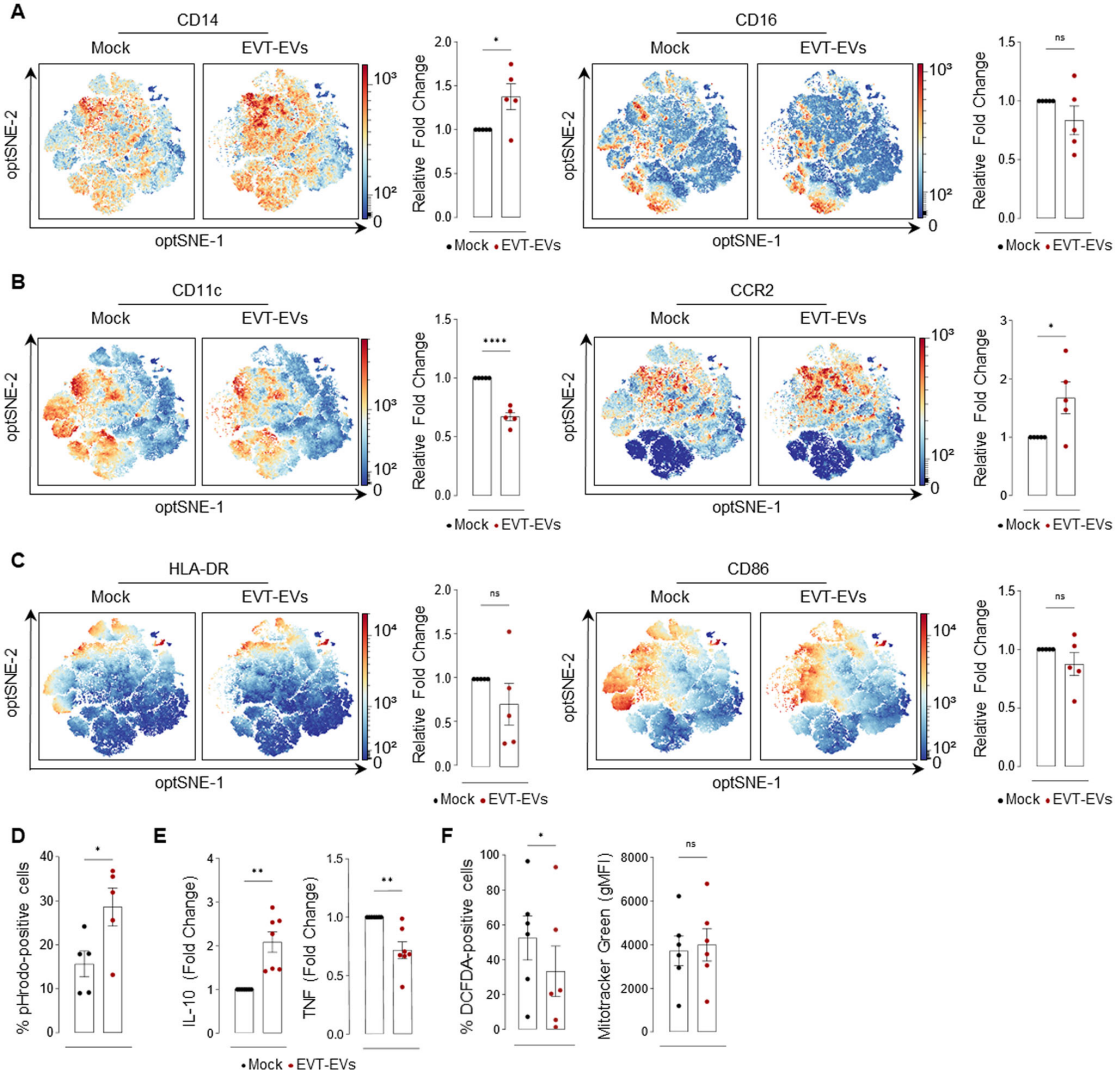
EVT‐EVs promote an anti‐inflammatory phenotype in monocytes. (A–C) opt‐SNE scatterplots (left) showing surface marker expression in mock‐ and EVT‐EVs‐treated monocytes, with corresponding bar plots (right) depicting fold‐change in geometric mean fluorescence intensity relative to mock. Color intensity reflects marker expression (deeper red = higher expression). (D–F) Functional assays of mock and EVT‐EVs–primed monocytes under LPS stimulation. (D) Phagocytic capacity, shown as the percentage of pHrodo‐positive cells. (E) IL10 and TNF secretion in culture supernatants (pg/mL), represented as relative fold‐change. (F) Reactive oxygen species (ROS) production (percentage of DCFDA‐positive cells) and mitochondrial mass (Mitotracker Green intensity). Data are mean ± SEM from independent experiments. Black circles indicate mock; red circles indicate EVT‐EV–primed monocytes. Statistical comparisons were performed using paired t‐tests. ns, not significant; p < 0.05 (*), p < 0.01 (**), p < 0.0001 (****).

Circulating monocytes are phenotypically and functionally classified into three subsets: classical CD14^+^CD16^−^ monocytes, characterized by high phagocytic capacity and robust migratory potential; intermediate CD14^+^CD16^+^ monocytes, the most pro‐inflammatory subset with potent antigen presentation and cytokines/ROS production; and non‐classical CD14^low^CD16^+^ monocytes, which patrol the endothelium and contribute to anti‐viral defense and tissue repair (Kapellos et al. 2019). To contextualize the proteomic shifts induced by EVT‐EVs education, we next examined key cell surface markers associated with monocyte phenotype and function. Multiparametric flow cytometry revealed that EVT‐EVs education significantly increased CD14 expression while decreasing CD16 levels, indicating a shift towards a more classical, less inflammatory monocyte phenotype (Figure 4A). This phenotype reprogramming was accompanied by reduced expression of CD11c, an integrin involved in cell adhesion, and upregulation of CCR2, a chemokine receptor that mediates monocyte recruitment to inflamed tissues (Figure 4B). EVT‐EVs‐treated monocytes exhibited a trend towards reduced expression of HLA‐DR and CD86 co‐stimulatory molecules both involved in antigen‐presentation, although these changes did not reach statistical significance (Figure 4C). Similar phenotypic reprograming was also observed with PHT‐EVs (Figure S2C). Functionally, upon lipopolysaccharide (LPS) stimulation, EVT‐EVs‐educated monocytes displayed enhanced phagocytic capacity (Figure 4D), increased production of the anti‐inflammatory interleukin‐10 (IL10) and reduced secretion of the pro‐inflammatory mediator tumour necrosis factor (TNF) (Figure 4E). Additionally, they generated lower levels of ROS while maintaining mitochondrial pool (Figure 4F), indicating preserved metabolic competence under stress conditions. Together, these data support a model in which EVT‐EVs reprogram monocytes toward a resilient regulatory‐leaning phenotype.

### 3.5 EVT‐EVs Promote Tolerogenic Antigen Presentation and Regulatory T Cell Expansion

**FIGURE 5:**
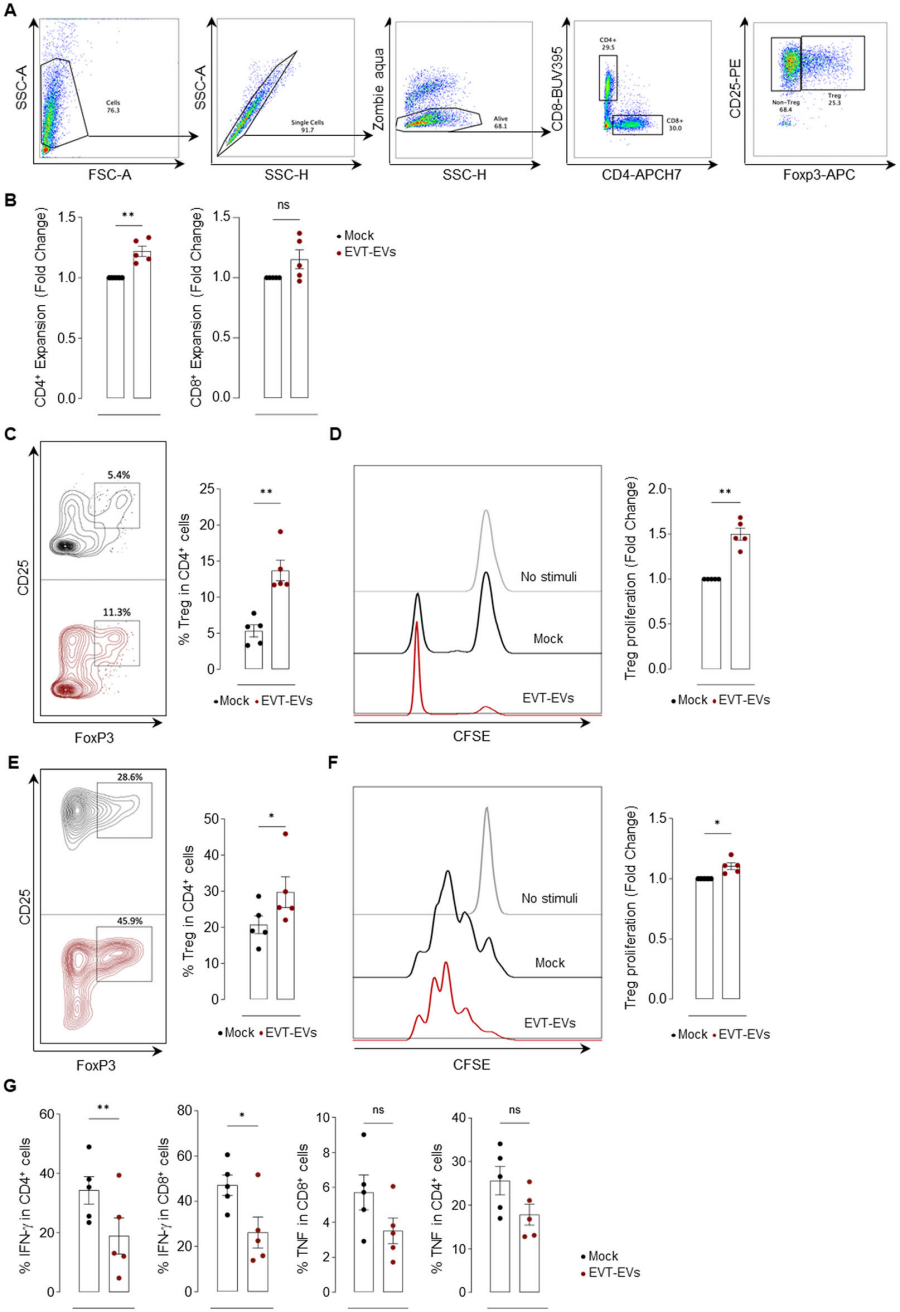
EVT‐EVs‐treated monocytes enhance regulatory T cell expansion and downregulate T cell responses. (A) Gating strategy. (B) Expansion of CD4^+^ and CD8^+^ T cells following co‐culture of HLA‐mismatched PBMCs with mock‐ or EVT‐EVs–treated monocytes shown as fold‐change. (C) Representative contour plots (left) and bar plot (right) showing the frequency of CD25^+^FoxP3^+^ regulatory T cells (Tregs) among CD4^+^ T cells. (D) Representative histograms (left) and bar plot (right) showing CFSE‐based proliferation and fold‐change in proliferation of CD4^+^CD25^+^FoxP3^+^ Tregs. (E) Representative contour plots (left) and bar plot (right) showing the frequency of CD25^+^FoxP3^+^ Tregs among CD4^+^ T cells following co‐culture with mock‐ or EVT‐EVs–treated monocytes in the presence of CD3/CD28/CD2 antibody complexes. (F) Representative histograms (left) and bar plot (right) showing CFSE‐based proliferation and fold‐change in proliferation of CD4^+^CD25^+^FoxP3^+^ Tregs under these conditions. (G) Frequency of IFN‐γ^+^ and TNF^+^ CD4^+^ and CD8^+^ T cells following co‐culture with mock‐ or EVT‐EVs–treated monocytes in the presence of CD3/CD28/CD2 antibody complexes. Data are mean ± SEM from independent experiments. Black circles indicate mock‐treated monocytes; red circles indicate EVT‐EVs–treated monocytes; “No stimuli” indicates unstimulated PBMCs. Statistical comparisons were performed using paired t‐tests. p < 0.05 (*), p < 0.01 (**).

Given that EVT‐EVs‐educated monocytes displayed a more immunomodulatory phenotype, we next investigated their capacity to influence adaptive T cell immune responses. HLA‐mismatched CFSE‐labelled PBMCs were co‐cultured with either EVT‐EVs‐treated or untreated monocytes for five days, then T‐cell proliferation was evaluated. Flow cytometry (Figure 5A) analysis showed that EVT‐EVs‐educated monocytes significantly enhanced CD4^+^ T cells expansion, with only a modest effect on CD8^+^ cells (Figure 5B). Notably, the expansion in CD4^+^ was largely driven by a marked proliferation of CD4^+^CD25^+^Foxp3^+^ regulatory T cells (Tregs), a key immunoregulatory subset that maintains peripheral tolerance and prevents autoimmune responses (Figure 5C,D).

To further evaluate the regulatory potential of EVT‐EVs‐educated monocytes, we activated CFSE‐labelled HLA‐mismatched PBMCs with CD3/CD28/CD2 antibody complexes in the presence of EVT‐EVs‐treated or untreated monocytes. Under these stimulatory conditions, EVT‐EVs–educated monocytes significantly increased both the frequency (Figure 5E) and proliferation (Figure 5F) of CD4^+^CD25^+^Foxp3^+^ Tregs. Concurrently, EVT‐EVs‐educated monocytes dampened the production of IFN‐γ, a key pro‐inflammatory cytokine, and tended to reduce TNF levels within both CD4^+^ and CD8^+^ T cell subsets (Figure 5G).

Altogether, these findings indicate that EVT‐EVs‐educated monocytes acquire a resilient, regulatory‐leaning phenotype that enable modulation of allogeneic T cell responses and promotes regulatory T cell expansion. While individual panels may show modest changes, the consistency across proteomic, pathway, and these functional analyses highlight the potential of EVT‐EVs as immunomodulatory agents capable of resolving inflammation through targeted reprogramming of innate antigen‐presenting cells.

### 3.6 EVT‐EVs Remodel the Monocyte Lipidome and Promote the Production Pro‐Resolving Lipid Mediators

**FIGURE 6:**
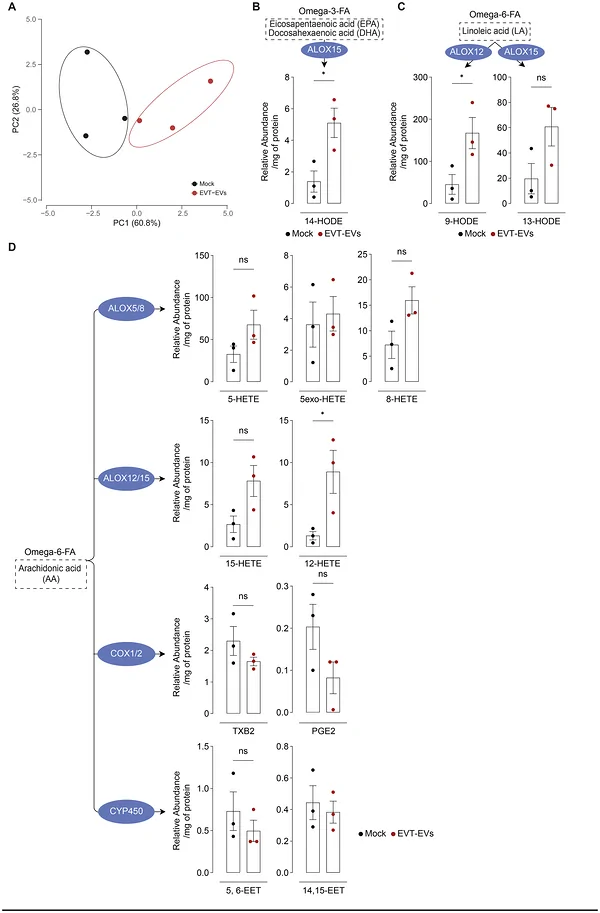
EVT‐EVs reshape monocyte lipidome and promote production of pro‐resolving lipid mediators. (A) Principal component analysis (PCA) of eicosanoid profiles from mock and EVT‐EVs‐treated monocytes. (B–D) Relative abundance of lipid mediators derived from docosahexaenoic acid (DHA) and eicosapentaenoic acid (EPA) (B), linoleic acid (LA) (C), and arachidonic acid (AA) (D). Data are presented as mean relative abundance per mg protein ± SEM from three independent experiments. Black circles indicate mock; red circles indicate EVT‐EVs‐treated monocytes. Statistical comparisons were performed using paired *t*‐tests. *p* < 0.05 (*).

As in other immune cells, monocyte behaviour and function, including activation, differentiation, migration, and inflammatory responses, are tightly regulated by lipids. Among these, eicosanoids, a class of bioactive lipid mediators derived from polyunsaturated fatty acids (PUFAs) such as arachidonic acid (AA), linoleic acid (LA), docosahexaenoic acid (DHA), and eicosapentaenoic acid (EPA), play critical roles in both initiating and resolving inflammation (Atalay Ekiner et al. 2024). Building on our proteomic data, which revealed enrichment of lipid metabolism pathways, including activation of nuclear liver X receptor (LXR) signaling and ATF4‐mediated stress responses, and predicted modulation of DHA‐derived lipid mediators (Figure 3), we next investigated whether EVT‐EVs education induces lipidomic reprogramming in monocytes. To this end, we performed targeted LC–MS/MS specifically designed to quantify bioactive eicosanoids and related lipid mediators’ landscape of EVT‐EVs‐treated versus untreated monocytes. PCA revealed distinct clustering between the two groups, indicating a substantial shift in lipid profiles upon EVT‐EVs exposure (Figure 6A). Quantitative analysis identified notable changes in twelve key eicosanoids. EVT‐EVs‐educated monocytes displayed a pronounced increase in 14‐HDoHE, an EPA/DHA‐derived precursor of specialized pro‐resolving mediators (SPMs) such as resolvins and protectins (Figure 6B). Levels of 9‐HODE and 13‐HODE, hydroxylated LA metabolites known to support inflammation resolution, were also elevated (Figure 6C). Additionally, EVT‐EVs exposure enhanced the abundance of AA‐derived 12‐HETE and 15‐HETE, both of which are associated with anti‐inflammatory and pro‐resolving activity. Notably, while pro‐resolving lipids were increased, levels of classical cyclooxygenase (COX)‐derived AA metabolites, TXB2 and PGE2, were reduced (Figure 6D). Certain non‐classical eicosanoids, such as 5,6‐EET and 14,15‐EET, were also downregulated. Although modest elevations were observed in certain AA‐derived eicosanoids with pro‐inflammatory potential, including 5‐HETE and 8‐HETE, the overall lipidomic profile was rather skewed toward resolution rather than propagation of inflammation.

Together, these results confirm and expand upon proteomics‐based predictions, demonstrating that EVT‐EVs actively reprogram the monocyte lipidome to favor the biosynthesis of pro‐resolving bioactive lipid mediators. This lipidomic shift validate and extend the model of EVT‐EVs‐driven monocyte reprogramming towards an anti‐inflammatory phenotype, contributing to broad immunomodulatory adaptation.

### 3.7 EVT‐EVs Modulate Inflammatory Monocytes in RDEB Patients

**FIGURE 7:**
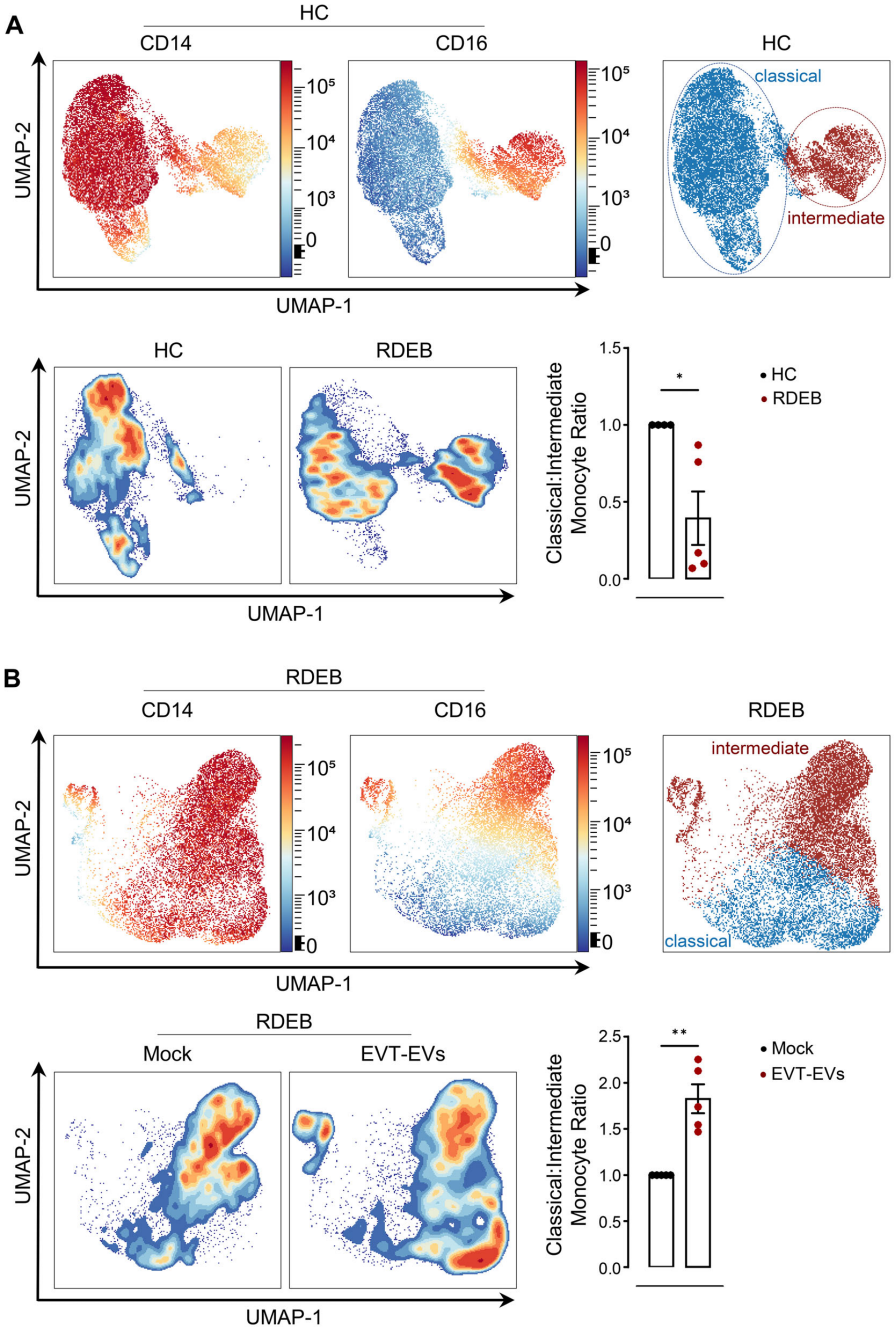
EVT‐EVs modulate inflammatory monocytes in patients with recessive dystrophic bullosa (RDEB). (A) Upper panel, UMAP of blood monocytes from healthy controls (HCs). Upper left, CD14 and CD16 expression; upper right, merged UMAP showing distribution of classical and intermediate monocytes. Lower panel left, density plot showing monocyte subset distribution in HCs (*n* = 4; black circles) and RDEB patients (*n* = 5; red circles). Lower right, bar plot showing the ratio of classical to intermediate monocytes in both groups. (B) UMAP of blood monocytes from RDEB patients. Upper left, CD14 and CD16 expression; upper right, merged UMAP showing distribution of classical and intermediate monocytes. Lower left, density plots showing distribution of subsets in mock‐ and EVT‐EV–treated RDEB monocytes. Lower right, bar plot showing the ratio of classical to intermediate subsets in mock (black circles) and EVT‐EVs–treated (red circles) monocytes. Color intensity reflects marker expression (deeper red = higher expression). Data are mean ± SEM. Statistical comparisons were performed using paired *t*‐tests. *p* < 0.05 (*), *p* < 0.01 (**).

Applying systems immunology approaches, we recently identified distinct inflammatory and immune signatures in adults with RDEB, including and increased frequency of pro‐inflammatory CD14^+^CD16^+^ intermediate monocytes and systemic pro‐inflammatory lipid profile (Hirt et al. 2025). Therefore, to assess the capacity of EVT‐EVs to modulate a pathological immune phenotype, we examined their effect on circulating monocytes from RDEB patients compared to healthy controls (HC). Uniform manifold approximation and projection (UMAP) analysis of whole blood was used to visualize monocyte subset distribution based on CD14 and CD16 expression (Figure 7A upper panel). Healthy individuals, monocytes displayed a distribution dominated by CD14^+^CD16^−^ classical monocytes with a minor population of CD14^+^CD16^+^ intermediate cells. In contrast, monocytes from RDEB patients showed a skewed distribution, with an elevated proportion of CD14^+^CD16^+^ intermediate relative to CD14^+^CD16^−^ classical monocytes (Figure 7A lower panel). Treatment of RDEB monocytes with EVT‐EVs for three days significantly restored this balance, increasing the proportion of CD14^+^CD16^−^ classical monocytes while reducing CD14^+^CD16^+^ intermediate pro‐inflammatory cells (Figure 7B).

These findings demonstrate that EVT‐EVs can reprogram inflammatory monocytes in RDEB toward a more homeostatic, less inflammatory phenotype, highlighting their potential as immunomodulatory agents in chronic inflammatory conditions.

## 4 Discussion

Intervention strategies that actively promote the resolution of inflammation are gaining recognition as effective approaches for managing a broad spectrum of inflammatory diseases. Central to this process is the dynamic reprogramming of monocyte and macrophage functions, enabling the transition from a pro‐inflammatory to a pro‐resolving immune landscape that support tissue repair. Here, using integrated proteomic and lipidomic analyses, we show that EVT‐EVs rewire immunometabolic pathways to promote a regulatory‐leaning phenotype in circulating monocytes. Although no single dominant mechanism was identified, the convergent multi‐omics and functional analysis reveal a coordinated immunoregulatory program induced by these EVs. Collectively, these findings position EVT‐EVs as modulators of innate immune plasticity and highlight their potential as a translational platform for resolution‐directed therapies aimed at restoring immune balance in patients with RDEB and potentially other chronic inflammatory disorders.

Extracellular vesicles are structurally complex entities carrying membrane‐bound and cytosolic proteins, along with small RNAs and miRNAs, enabling them to modulate the behaviour of recipient cells. Our proteomic analysis revealed that EVT‐EVs reconfigure the monocyte proteome toward a regulatory‐leaning phenotype, that is immunologically active yet tightly regulated favouring the resolution of inflammation and supporting tissue repair. Immune profiling indicated that EVT‐EVs display a hypoimmunogenic signature, marked by the expression of HLA‐G and PD‐L1, molecules associated with immune tolerance and regulatory T cell (Treg) expansion (Bu et al. 2021; Burke et al. 2024). Upon exposure to EVT‐EVs, monocytes acquired tolerogenic properties and supported the proliferation of CD4^+^CD25^+^Foxp3^+^ Tregs, both in allogeneic settings and during active immune responses. Consistent with this, EVT‐EVs‐educated monocytes exhibited reduced surface expression of HLA‐DR and CD86 compared to unexposed cells, aligning with a phenotype typical of tolerogenic antigen‐presenting cells. This is supported by previous reports showing that tolerogenic dendritic cells, which promote Treg induction and expansion, typically display an immature phenotype with dimensioned expression of MHC class II and costimulatory CD80 and CD86 molecules (Drakul et al. 2023; Fisher et al. 2024).

Direct evidence supporting the role of HLA‐G‐ and PD‐L1‐bearing EVs in inducing tolerogenic, immunosuppressive myeloid cells has been established in tumour immunology and immunotherapy context (Li et al. 2021; Lu and Yang 2024). For instance, glioblastoma‐derived EVs expressing PD‐L1 have been shown to promote the development of immunosuppressive non‐classical monocytes that inhibit T cell proliferation in vitro (Himes et al. 2020). Similarly, EVs released by renal cancer stem cells and carrying HLA‐G disrupt monocyte maturation and diminish their capacity to stimulate T cells (Grange et al. 2015). In another context, PD‐L1‐bearing EVs from Wharton's jelly‐derived mesenchymal stem cells have demonstrated efficacy in suppressing T cell activation in patients with acute graft‐versus‐host disease (Li et al. 2021). While the immunoregulatory functions of HLA‐G and PD‐L1 are well established, the mechanisms through which their EV‐associated forms exert these effects remain incompletely defined. One proposed mechanism involves direct engagement with inhibitory receptors, (ILT2/LILRB1/CD85j, ILT4/LILRB2/CD85d, and PD‐1), on recipient immune cells or internalization of EVs may activate intracellular signaling cascades involved in immune regulation (Rebmann et al. 2016). Beyond PD‐L1 and HLA‐G expression, our analysis revealed that EVT‐EVs are enriched in miRNAs with immunoregulatory proprieties. Notably, the let‐7 and miR‐154 families, which were highly enriched in our EVT‐EVs, are known to downregulate pro‐inflammatory transcripts while promoting anti‐inflammatory gene expression, supporting the acquisition of a regulatory monocyte phenotype (Baer et al. 2016; Lv et al. 2024; Yang et al. 2024). Additionally, miR‐29, miR‐23/‐27/24, and miR‐130 families have been implicated in modulating PD‐L1 expression and promoting Treg‐supportive functions in monocytes (Bai et al. 2022; Bulati et al. 2020; Luo et al. 2024), highlighting a broader miRNA landscape which likely acts as molecular switches reinforcing tolerance programs.

Reactive oxygen species (ROS) are critical signaling molecules involved in the pathogenesis of inflammatory disorders. Mitochondria are the primary source of ROS, and their dysfunction can compromise cellular homeostasis, leading to oxidative stress and tissue damage (Kowalczyk et al. 2021). Ingenuity pathway analysis of our proteomic data revealed that EVT‐EVs modulate pathways associated with mitochondrial dysfunction, nitric oxide (NO) and ROS generation, while concurrently upregulating antioxidant defense mechanisms. In line with this, EVT‐EVs enhanced the expression of superoxide dismutase 2 (SOD2), a mitochondrial enzyme essential for detoxifying superoxide radicals, indicating reinforcement of antioxidant defenses and improved resilience to oxidative stress. Functionally, EVT‐EVs‐educated monocytes produced less ROS while preserving mitochondrial mass, reflecting enhanced cellular resilience under inflammatory stress. These redox adaptations also influence extracellular matrix (ECM) dynamics (Martins et al. 2021) and may be particularly relevant in RDEB, where oxidative damage and mitochondrial dysfunction compromise skin integrity and wound healing.

Beyond antioxidant, EVT‐EVs induced broad chromatin remodelling, reflected by the upregulation of core histones (H2AC25, H2AC20, H2BC21, and H3). Increased nucleosome assembly and epigenetic plasticity may enable transcriptional flexibility, supporting the transition toward anti‐inflammatory and tissue‐repair programs. Such chromatin reconfiguration has been implicated in the acquisition of tolerogenic monocyte states and may represent a durable mechanism through which EVT‐EVs stabilize regulatory immunity.

Proteomic profiling also revealed the downregulation of ITIH1 and ITIH2, acute‐phase proteins typically elevated during inflammation, as well as suppression of CLU metabolism, together indicating reduced pro‐inflammatory metabolic drive. Conversely, monocytes upregulated enzymes involved in NAD^+^ metabolism (NAMPT), kynurenine pathway activity (KYNU), oxidative phosphorylation (ATP1B3), and fatty acid activation (ACSL1), highlighting a shift toward metabolic flexibility and production of immunoregulatory metabolites. Such metabolic rewiring is increasingly recognized as a cornerstone of monocyte plasticity, linking cellular energetics to functional phenotype.

EVT‐EVs further reinforced activation, phagocytic and immune recognition pathways. We observed upregulation of CD14, MRC1 (CD206), FCN1, as well as TLR2, molecules central to microbial sensing and clearance (Freeman and Grinstein 2014; Gordon 2016; Na et al. 2023). Functionally, these changes correlated with enhanced phagocytic activity in EVT‐EVs‐treated monocytes, which is highly relevant for RDEB patients, where recurrent infections perpetuate inflammation and impair healing. At the same time, induction of interferon‐stimulated genes (ISG15, IFIT1, IFIT2, IFIT3) suggested a state of antiviral readiness, ensuring pathogen defense without provoking excessive inflammation. Regulation of serpin family proteins, including downregulation of SERPIN A3 and G1 and upregulation of SERPIN B9 and CD59, further underscores the immune recalibration induced by EVT‐EVs, supporting cytoprotection and immune balance.

Our proteomic analysis also highlighted key regulatory pathways associated with lipid metabolism and cellular stress adaptation, including nuclear liver X receptor (LXR) activation, an important transcriptional regulator of cholesterol and fatty acid metabolism, and ATF4‐mediated stress responses, which coordinate metabolic homeostasis under stress conditions (Guo et al. 2021; Hotamisligil and Bernlohr 2015; Korner et al. 2019; Mukherjee et al. 2020). EVT‐EVs treatment of monocytes led to increased levels of hydroxylated linoleic acid (LA) metabolites, including both 9‐HODE, typically linked to pro‐inflammatory signaling, and 13‐HODE, known for its anti‐inflammatory properties (Tam 2013), indicating a dual modulatory effect. Importantly, 14‐HDoHE, a precursor to specialized pro‐resolving mediators (SPMs) that limit immune cell infiltration and support tissue homeostasis, was considerably elevated. Additionally, anti‐inflammatory AA‐derived metabolites such as 12‐HETE and 15‐HETE were increased HETE (Kronke et al. 2009). While some pro‐inflammatory AA metabolites, including 5‐HETE and 8‐HETE, were also increased, EVT‐EVs markedly reduced COX‐derived products, TXB_2_ and PGE_2_, which are typically upregulated in inflammatory conditions and promote Th17 differentiation via EP2 and EP4 receptor signaling (Boniface et al. 2009). This lipidomic reprogramming shifts the monocyte eicosanoid landscape toward a pro‐resolving, immunoregulatory phenotype (Chiurchiu et al. 2018).

Mechanistically, the coordinated changes in chromatin, metabolism, antioxidant defenses, phagocytosis, interferon signaling, and lipid mediators collectively highlight EVT‐EVs as a resilience‐promoting platform, enabling monocytes to maintain functional competence under stress while avoiding hyperinflammation. Our integrated pathway and network analyses suggest that EVT‐EVs act as an integrated regulatory unit, coupling mitochondrial, epigenetic, and metabolic reprogramming with immunomodulatory output, a principle reminiscent of trophoblast‐mediated maternal tolerance.

Although this study does not yet identify the specific miRNAs, lipids, or proteins mediating individual EVT‐EV effects, convergent multi‐omics and functional analyses support a coherent immunoregulatory program and provide a framework for future preclinical evaluation of placental EVs as modulators of innate immune function. Notably, a double‐blind randomized controlled trial proposed human placental mesenchymal stromal cell‐derived small extracellular vesicles as a possible treatment for critically ill patients with COVID‐19 (Zamanian et al. 2024), highlighting the translational potential of placental EVs in modulating systemic inflammation. In line with this paradigm, our integrated pathway and network analyses support a model in which EVT‐EVs reprogram circulating monocytes into a metabolically active, immunoregulatory state marked by antiviral readiness, redox balance, and enhanced resilience to cellular stress, all while circumventing overt inflammation.

While SV40 large T antigen–immortalized HIPEC cells provide a promising surrogate system for generating a standardized, reproducible, and scalable source of EVT‐derived EVs, immortalization may influence cellular transcriptional programs and, consequently, EV cargo composition. Importantly, the integrated SV40 large T antigen present in these cells does not include the full SV40 genome (Pavan et al. 2003) and therefore cannot support viral replication, late gene expression, or virion assembly, processes that require an intact viral genome (Sullivan and Pipas 2002). As a result, the presence of intact SV40 viral particles or structural proteins in EVT‐EV preparations is highly unlikely. Similarly, expression of SV40‐derived microRNAs is improbable, as the only well‐characterized SV40 microRNA (miR‐S1‐5p/3p) is encoded within the viral late region and requires the complete viral genome during productive replication. Nevertheless, the possibility that EVT‐EVs may contain transformation‐associated molecular signatures cannot be fully excluded. Accordingly, future translational development should consider the potential influence of SV40‐driven immortalization on EV cargo composition and will require rigorous safety evaluation prior to clinical application.

In summary, our findings position EVT‐EVs as a robust mechanistic and translational discovery platform. With further validation and rigorous safety profiling, EVT‐EVs may support the development of clinically deployable therapeutics capable of promoting immune homeostasis, enhancing stress resilience, and facilitating tissue repair in inflammatory disorders such as RDEB and potentially other inflammatory conditions. Collectively, these results highlight EVT‐EVs as a versatile platform for advancing both mechanistic insight and next‐generation EV‐based therapeutics.

## Author Contributions

**Nell Hirt**: investigation, methodology, writing – original draft, validation, formal analysis, visualization, data curation. **Enzo Manchon**: investigation, methodology, validation, formal analysis, writing – original draft, data curation, visualization. **Ming Wu**: visualization, software, formal analysis, data curation, methodology. **Helene Le Buanec**: formal analysis, validation. **Pauline Bataille**: resources. **Emmanuelle Bourrat**: resources, validation, writing – review and editing. **Jean‐David Bouaziz**: supervision, validation, writing – review and editing, resources. **Reem Al‐daccak**: supervision, writing – original draft, writing – review and editing, conceptualization, validation, visualization, project administration, funding acquisition. **Nabila Jabrane‐Ferrat**: supervision, conceptualization, validation, writing – review and editing, visualization, project administration, funding acquisition, writing – original draft.

## Funding

The work was supported by AO MARS 2020‐la Société Française de Dermatologie, AP‐RM‐22‐006‐Fondation d'Avenir, MEDIMACS‐ANR‐17‐HDIM‐0005‐02, MetaboHUB ANR‐11‐INBS‐0010, Fondation pour la Recherche Médicale ENV202003011510, Inserm grants for UMRS1342 and Inserm‐CNRS‐University of Toulouse recurrent funds to R.D. and N.J.F. M.W. is a recipient of the Chinese Scholarship Council PhD fellowship.

## Ethics Statement

All protocols were approved by the institution (CPP No. 2023‐A01351‐44) and were conducted in accordance with guidelines from the Declaration of Helsinki.

## Consent

All participants signed informed consent following human ethics committee “Comité consultatif pour la protection des personnes dans les recherches biomédicales” (Direction de la Recherche Clinique, de l'Innovation des relations avec les universités et les organismes de recherche (DRCI), AP‐HP, Saint‐Louis Hospital).

## Conflicts of Interest

The authors declare no conflict of interest.

## Supporting information




**Supporting material**: jev270315‐sup‐0001‐SuppMat.docx

## Data Availability

All data generated and analyzed are included in the current study and are available from the corresponding authors on reasonable request.
