# Supplementary material for: Trophoblast Extracellular Vesicles Rewire Immunity: A Symptom‐Relief Avenue for Recessive Dystrophic Epidermolysis Bullosa
Source: J Extracell Vesicles. 2026 Sep 24;15(10):e70315. doi: 10.1002/jev2.70315 (PMC13603074; doi:10.1002/jev2.70315)
Supplement: Supplementary file 1 [file JEV2-15-e70315-s001.docx]

**Trophoblast Extracellular Vesicles Rewire Immunity: A Symptom-Relief Avenue for Recessive Dystrophic Epidermolysis Bullosa**

Nell Hirt^1†^, Enzo Manchon^1†^, Ming Wu^2†^, Helene Le Buanec^1^, Pauline Bataille^3^, Emmanuelle Bourrat^3^, Jean-David Bouaziz^1,3††^, Nabila Jabrane-Ferrat^2††*^ and Reem Al-Daccak^1††*^

^1^University of Paris, INSERM, CNRS, IRSL – Saint Louis Research Institute, Saint-Louis Hospital, Paris, France.

^2^University of Toulouse, CNRS, INSERM, Infinity – Institute for Infectious and Inflammatory Diseases, Toulouse, France.

^3^AP-HP, Department of Dermatology, Saint-Louis Hospital; 75010 Paris, France.

**Supplementary material and Figures**

^†^These authors contributed equally and share first-authorship.

^††^These authors jointly supervised the work.

*Corresponding authors:

Reem Al-Daccak, PhD

Saint-Louis Research Institute, IRSL

1, Avenue Claude Vellefaux, 75010, Paris - France

email: [reem.al-daccak@inserm.fr](mailto:reem.al-daccak@inserm.fr)

Nabila Jabrane-Ferrat, PhD

Institute for Infectious and Inflammatory Diseases, Infinity

CHU Purpan, Toulouse, France

email: [nabila.jabrane-ferrat@inserm.fr](mailto:nabila.jabrane-ferrat@inserm.fr)

**Supplementary Methods**

**Primary human trophoblast extracellular vesicles**

The study was approved by the French South-West & Outre-Mer II Ethics Committee and registered with the French Ministry of Higher Education and Research (DC-2016-2772). First-trimester placental tissues (7–11 weeks of gestation) were obtained from healthy women undergoing elective termination of pregnancy after written informed consent, in accordance with the Declaration of Helsinki.

Primary human trophoblast (PHT) explants were cultured in DMEM/F12 (1:1, v/v) supplemented with extracellular vesicle (EV)-depleted fetal bovine serum for 72 h. PHT-derived EVs (PHT-EVs) were isolated from clarified culture supernatants by differential ultracentrifugation according to standardized protocols, in compliance with the latest International Society for Extracellular Vesicles guidelines. Isolated PHT-EVs were stored at −80 °C until use.

**Table 1: Antibodies used in this study**

| **Antibody** | **Clone** | **Source** | **Cat # identifier** |
| --- | --- | --- | --- |
| CD14-PE | REA599 | Miltenyi | 130-110-577 |
| CD16-BV711 | 3G8 | BD Bioscience | 563127 |
| CD11c-FITC | B-Iy6 | BD Bioscience | 561355 |
| CCR2-APC | K036C2 | Biolegend | 357208 |
| HLA-DR-BV421 | G46-6 | BD Bioscience | 562804 |
| CD86-PECF594 | 2331 | BD Bioscience | 562390 |
| CD25-PE | M-A251 | BD Bioscience | 555432 |
| FoxP3-APC | 236A/E7 | Invitrogen | 17-4777-42 |
| CD4-APCH7 | RPA-T8 | BD Bioscience | 560158 |
| CD8-BV395 | RPA-T8 | BD Bioscience | 563795 |
| CD163-BV421 | GHI/61 | BD Bioscience | 562643 |
| CD36-BV395 | CB38 | BD Bioscience | 565422 |
| CD64-PECY7 | RE978 | Miltenyi | 130-116-301 |
| CD206-APC | 19.2 | BD Bioscience | 561763 |
| IFNγ-PECY7 | B27 | BD Bioscience | 557643 |
| TNF-PE | MAb11 | BD Bioscience | 559321 |

**Supplementary Figures and Legends**

**Figure S1. PHT-derived extracellular vesicles (PHT-EVs) exhibit an immunomodulatory signature.**

(A) Nanoparticle tracking analysis (NTA) showing size distribution of PHT-EVs.

(B) Western blot of CD63, syntenin-1, PAP, TSG101 and ADAM10 in parental EVT lysates, EVT-EVs and PHT-EVs. (C) Surface protein expression profile presented as log₁₀-transformed geometric mean intensity in PHT-EVs. (D) Left panel: Scatter plot showing the relationship between miRNA family-level distribution between PHT-EVs and EVT-EVs. Each point represents a miRNA family. The y-axis reports the family mean score in PHT-EVs and the x-axis reports the family mean score in EVT-EVs. Bubble size is proportional to the number of specific miRNAs within each family. Pearson’s correlation coefficient quantifies overall similarity across families. Right panel: Bubble plot showing the relative weighted value of shared miRNA families between PHT-EVs and EVT-EVs. Bubble size reflects the weighted mean value for each family. Positive values are shown in red, negative values in blue.

**Figure S2.** (A) **CFSE-labelled EVT-EVs purity and dye exclusion.** Nanoparticle tracking analysis (NTA) showing size distribution of CFSE-labelled EVT-EVs. (B) **TLR2** **expression in PHT-EVs and EVT-EVs**. Bar plot showing the relative abundance of TLR2 protein levels. (C) **PHT-EVs promote an anti-inflammatory phenotype in monocytes**. Bar plots showing fold-change in geometric mean fluorescence intensity of surface markers in Mock- and PHT-EVs-treated monocytes. *p* < 0.05 and fold change > 1.5 are considered significant. Not significant (ns); p < 0.05 (*).
